# Factors influencing preconception care awareness and knowledge among women in Africa: a systematic review

**DOI:** 10.3389/frph.2025.1702378

**Published:** 2026-01-09

**Authors:** Patience Fakornam Doe, Amidu Alhassan, Boahemaa Adu Otchere, Frank Offei Odonkor, Isaac Aidoo Erzuah, Yvonne Dorothy Mintah, Hilda Kessewah Koranteng, Mustapha Amoadu

**Affiliations:** 1 Department of Public Health, School of Nursing and Midwifery, College of Health and Allied Sciences, University of Cape Coast, Cape Coast, Ghana; 2 Department of Adult Health, School of Nursing and Midwifery, College of Health and Allied Sciences, University of Cape Coast, Cape Coast, Ghana; 3 School of Medical Sciences, College of Health and Allied Sciences, University of Cape Coast, Cape Coast, Ghana; 4 Biomedical and Clinical Research Centre, College of Health and Allied Sciences, University of Cape Coast, Cape Coast, Ghana

**Keywords:** preconception care, maternal health, knowledge, risk factors, protective factors

## Abstract

**Background:**

Preconception care (PCC) plays a critical role in enhancing maternal and neonatal health by addressing risk factors before pregnancy. Most existing reviews focus on PCC outcomes such as utilization with limited attention to factors influencing PCC awareness and knowledge. This review addressed this gap by synthesizing evidence on PCC awareness and knowledge levels and by examining the associated risk and protective factors among women in Africa.

**Methods:**

The review was guided by the Preferred Reporting Items for Systematic Reviews and Meta-Analyses (PRISMA) guidelines. Five main databases (PubMed Central, African Journals Online, Web of Science, Scopus, and Journal Storage) were searched in March 2025, and 27 articles met the eligibility criteria for inclusion. The Joanna Briggs Institute critical appraisal checklist was used to assess the methodological quality of the studies. Data were analyzed using a narrative review approach to evaluate awareness and knowledge levels, as well as the risk and protective factors influencing PCC.

**Results:**

Awareness levels of PCC ranged from 5.9% in Ethiopia to 91% in Tanzania, while knowledge levels varied from 11% in Sudan to 70% in Tanzania. Low awareness and knowledge were associated with risk factors such as low education, poor socioeconomic status, limited healthcare access, lack of antenatal care (ANC), and adverse pregnancy histories. Protective factors contributing to higher PCC awareness and knowledge included higher educational attainment, greater economic stability, more frequent ANC visits, greater media exposure, and improved access to counseling and support services.

**Conclusion:**

Despite encouraging progress in some regions, significant gaps in PCC awareness and knowledge remain across African countries, particularly in low-resource settings. Bridging these gaps will require context-specific educational strategies, policy support, and expanded access to quality healthcare services tailored to African health systems.

**Systematic Review Registration:**

https://doi.org/10.17605/OSF.IO/VPWZG

## Introduction

Preconception care (PCC) refers to a set of health interventions designed to optimize maternal well-being before conception to improve pregnancy outcomes (1). It involves health promotion, risk assessment, disease prevention, and medical interventions that aim to address factors affecting fertility, maternal health, and fetal development (2). PCC plays a crucial role in maternal and child health by reducing adverse pregnancy outcomes such as preterm birth, congenital anomalies, and maternal complications (3). The key objectives of PCC include ensuring adequate maternal nutrition, managing pre-existing health conditions, preventing infections, and promoting positive health behaviors that contribute to a healthy pregnancy (1, 4). However, inadequate PCC often results in missed opportunities to modify risk factors before conception, leading to preventable non-communicable disease exposures, teratogenic effects, and untreated infections (5, 6). Consequently, women face higher risks of preterm birth, stillbirth, and congenital anomalies, while newborns are more likely to experience low birth weight and neonatal mortality (7, 8).

Global statistics highlight the urgency of PCC, with the WHO reporting that 74 million women living in low and middle-income countries have unintended pregnancies annually, leading to increased maternal and neonatal risks (9). In sub-Saharan Africa, pooled prevalence estimates from recent studies reveal that only approximately 33.27% of women of reproductive age demonstrate good knowledge of PCC (10) and that just 24.05 % utilize PCC services (10). The Centers for Disease Control and Prevention (CDC) advocates for PCC integration into primary healthcare to address gaps in pre-pregnancy health interventions (11, 12). Although developed within the US context, these guidelines are relevant to African health systems as they emphasize preventive, community-based, and multidisciplinary approaches that can be adapted to strengthen reproductive healthcare delivery in low-resource settings (11, 12). However, barriers such as limited healthcare infrastructure, financial constraints, and lack of awareness hinder access to PCC services (3).

PCC aligns with the United Nations Sustainable Development Goal (SDG) 3, which aims to ensure healthy lives and promote well-being for all ages. Specifically, target 3.1 seeks to reduce global maternal mortality to below 70 per 100,000 live births by 2030 (13). The current global maternal mortality ratio is approximately 197 deaths per 100,000 live births (14), while SDG 3.2 targets the elimination of preventable deaths among newborns and children under five. The current global maternal mortality ratio is approximately 197 deaths per 100,000 live births, with sub-Saharan Africa accounting for approximately 70% of these deaths (182,000) (14). These stark disparities underscore the urgent need for context-specific strategies to accelerate progress toward achieving the SDG target across African countries. Complementary frameworks such as the African Union's Maputo Plan of Action (15) and the WHO’s Reproductive, Maternal, Newborn, Child, and Adolescent Health (RMNCH) (16, 17) strategy reinforce these objectives by promoting equity, early intervention, and continuity of care. By integrating PCC into maternal health services, preventable causes of maternal and neonatal mortality can be addressed (1, 18). Research suggests that PCC interventions significantly improve maternal health outcomes and reduce pregnancy-related complications, particularly in low-resource settings (2). While these findings indicate substantial progress in promoting maternal and newborn survival, the determinants of PCC awareness and knowledge remain poorly characterized; therefore, strengthening PCC implementation is crucial for achieving global maternal and child health targets.

Effective PCC strategies focus on nutritional optimization, ensuring women achieve adequate nutrient levels before pregnancy to prevent deficiencies linked to adverse outcomes such as neural tube defects (19). Other essential interventions include infection prevention and treatment, family planning services, screening for chronic diseases such as diabetes and hypertension, and substance abuse prevention (5, 20, 21). Studies have demonstrated that women who receive PCC interventions are more likely to have improved pregnancy outcomes, including reduced risks of stillbirth, low birth weight, and maternal complications (22, 23). Despite this, awareness and knowledge of PCC remain low in many African countries, leading to missed opportunities for early interventions (10). In Africa, awareness and knowledge of PCC remain low, largely due to limited integration within healthcare systems (24). Studies indicate that many African women lack awareness of key PCC interventions, with knowledge influenced by factors such as age, education, family planning history, previous adverse birth outcomes, and the presence of chronic diseases (2, 3). Addressing these gaps through awareness campaigns and integrating PCC into routine maternal healthcare can significantly improve reproductive health outcomes.

Despite growing recognition of PCC's importance, knowledge gaps persist regarding the reasons behind disparities in PCC awareness (25). Several systematic reviews and meta-analyses have examined PCC and its influence on pregnancy and birth outcomes, highlighting its effectiveness in reducing maternal and neonatal morbidity and mortality (4, 10, 20, 26–29). However, while some reviews have examined preconception care in African settings such as Ethiopia (29, 30), they primarily focus on prevalence and utilization rates without delving into broader sociocultural, structural, and health system-related determinants. This review addresses these gaps by synthesizing evidence across Africa on PCC awareness and knowledge levels and explores the associated risk and protective factors. Moreover, existing literature has predominantly focused on the outcomes of PCC interventions rather than the underlying factors influencing PCC awareness and knowledge among women in Africa (2, 25). This limitation presents a critical gap, as sociocultural, economic, and healthcare system-related factors significantly shape health-seeking behaviors in African populations. However, filling this research gap is crucial, as maternal and neonatal health continue to pose significant public health challenges in Africa, where maternal mortality rates are among the highest globally (18). This study examined the factors influencing preconception care awareness and knowledge among women in Africa through a systematic review of existing literature. The findings served as a foundation for enhancing maternal healthcare policies and developing targeted interventions that promote PCC awareness and uptake in African settings. The evidence from this review will inform policymakers, healthcare providers, and stakeholders about the key factors influencing preconception care awareness and knowledge among women in Africa, enabling targeted interventions to improve maternal and neonatal health outcomes. Ultimately, it will provide a comprehensive understanding of the barriers and facilitators to PCC uptake, guiding future research and policy development in low-resource settings.

## Methods

This systematic review was conducted in accordance with the Preferred Reporting Items for Systematic Reviews and Meta-Analyses (PRISMA) guidelines (31). The review process encompassed formulating research questions, identifying and selecting relevant studies, extracting and summarizing data, synthesizing findings, reporting results, and engaging in consultation. The protocol has been successfully registered on Open Science Framework with DOI: https://doi.org/10.17605/OSF.IO/VPWZG.

### Review questions

The review was guided by two main research questions:

1. What is the level of awareness and knowledge of preconception care among women of reproductive age in Africa?
2. What factors influence the awareness and knowledge of preconception care among women in Africa?

### Search strategies

The search for relevant studies was conducted across five major databases, which include PubMed Central, African Journals Online (AJOL), Web of Science, Scopus, and Journal Storage. To ensure comprehensive literature coverage, we performed additional searches in Google Scholar and ProQuest. The initial search was conducted in PubMed using a combination of Medical Subject Headings (MeSH) terms and free-text keywords related to preconception care, awareness, knowledge, and influencing factors (Table 1). These search terms were developed based on the PICO (patient/problem, intervention, comparison, and outcome) framework and were subsequently refined to suit the search requirements of other databases. Boolean operators (AND, OR) were applied to enhance the precision and relevance of search results. To ensure a robust search strategy and effective data management, we consulted a chartered librarian. The final search across all databases was completed on 4 March 2025.

**Table 1:** Search strategy.

Database	Search strategy
PubMed Central via NCBI	(“preconception care”(MeSH Terms) OR “pre-pregnancy care” (All Fields) OR “preconception health” (All Fields)]) AND (“awareness” (All Fields) OR “knowledge”(All Fields) OR “determinants” (All Fields)) AND [“Africa”(All Fields) OR “Sub-Saharan Africa”(All Fields) OR “Ethiopia”(All Fields) OR “Nigeria"(All Fields) OR “Ghana” (All Fields)] AND (“2000/01/01” (PubDate): “2025/05/31” (PubDate)
Scopus	TITLE-ABS-KEY[“preconception care” OR “pre-pregnancy care” OR “preconception health”) AND TITLE-ABS-KEY(“awareness” OR “knowledge” OR “determinants”] AND TITLE-ABS-KEY(“Africa” OR “Sub-Saharan Africa” OR “Ethiopia” OR “Nigeria” OR “Ghana”) AND PUBYEAR > 1999 AND PUBYEAR < 2026
Web of Science	TS=(“preconception care” OR “pre-pregnancy care” OR “preconception health”) AND TS=(“awareness” OR “knowledge” OR “determinants”) AND TS=(“Africa” OR “Sub-Saharan Africa” OR “Ethiopia” OR “Nigeria” OR “Ghana”) AND PY=(2000–2025)
AJOL	(“preconception care” AND (“awareness” OR “knowledge” OR “determinants”) AND (“Africa” OR “Sub-Saharan Africa” OR “Ethiopia” OR “Nigeria” OR “Ghana”))—no filters applied; all publication years included
Dimensions AI	(“preconception care awareness” OR “pre-pregnancy care knowledge”) AND (“Africa” OR “Sub-Saharan Africa” OR “Ethiopia” OR “Nigeria” OR “Ghana”)—filter: English language only; all years
Google Scholar	“preconception care awareness” + “knowledge” + “Africa” + “determinants”—first 200 results screened; duplicates removed; English only; all publication years

### Study selection

**Figure 1:**
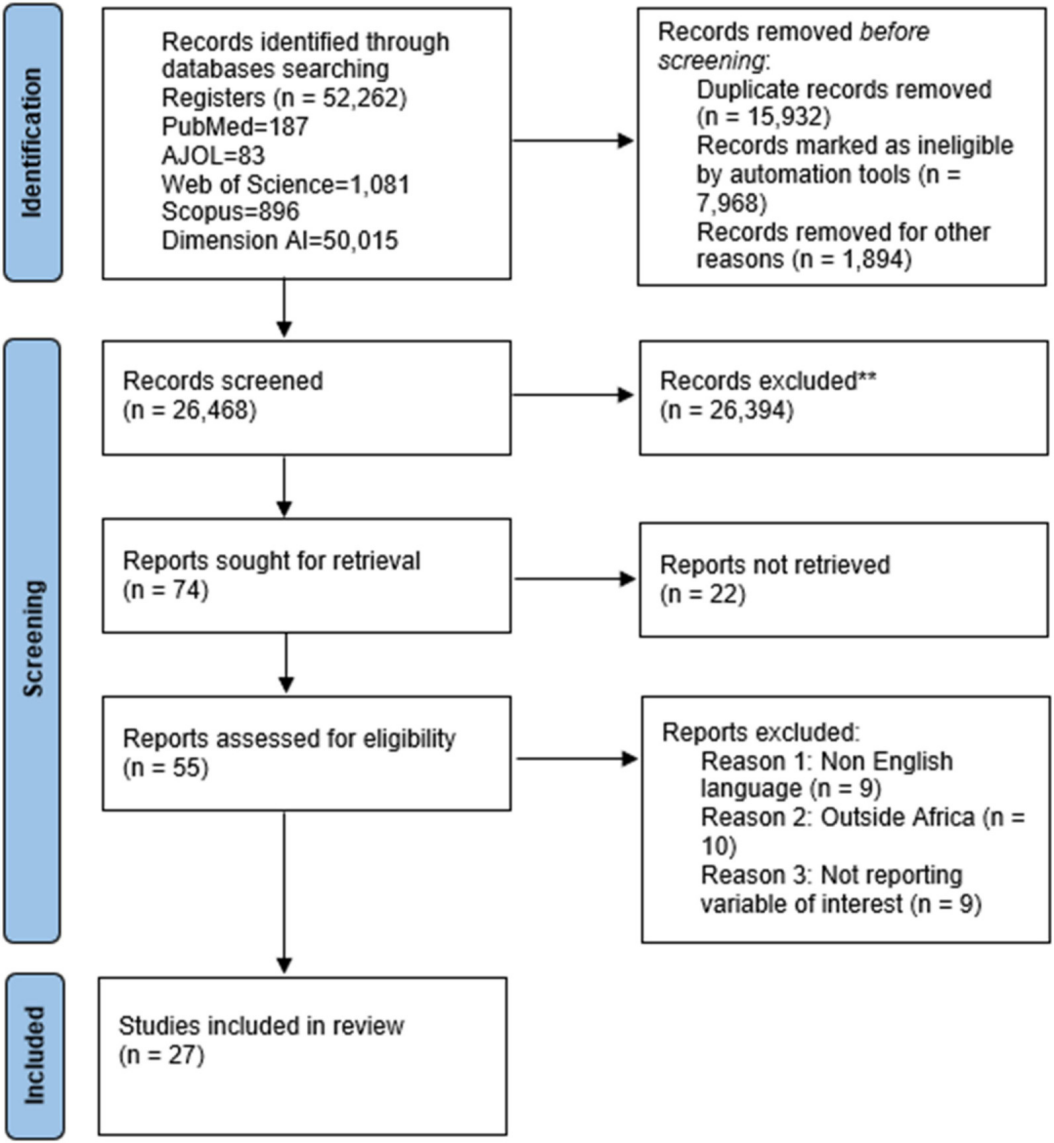
PRISMA flowchart.

Search results were imported into Mendeley reference management software, where the duplicate records were detected and removed. The study selection process was done in three stages. In the first stage, titles and abstracts of all studies yielded were screened for relevance by two independent reviewers under the supervision of the authors (MA, PD). At the second level, full-text versions of studies that were deemed potentially relevant were retrieved and compared with the prespecified eligibility criteria. The lists of references to the included full-text articles were also scanned to ascertain any further potentially relevant studies. At the final level, two reviewers independently conducted the full-text screening, where disagreements were handled by discussion between the two or consultation with a third reviewer if needed. Eligibility criteria employed using this process have been outlined in Table 2, while an overview of screening and results is described using the PRISMA flowchart shown in Figure 1.

**Table 2:** Inclusion and exclusion criteria.

Inclusion
Women of reproductive age (15–49 years) in African countries Studies examining awareness, knowledge, and factors influencing PCC Observational studies (cross-sectional, cohort, case-control), qualitative studies, mixed-methods research Studies conducted in African countries Full-text articles available Grey literature (dissertation or thesis) Papers published in the English language
Exclusion
Opinion pieces, reviews, commentaries, case reports, conferences, preprints, abstracts, or editorials Studies conducted outside Africa Studies conducted in any other language apart from English

### Appraisal of studies

The Joanna Briggs Institute (JBI) Critical Appraisal Tools (2020) were employed to assess the methodological quality of the included studies (32). For mixed-methods studies, the JBI checklist consists of 10 items assessing the appropriateness of the research design, rationale for using mixed methods, integration of qualitative and quantitative data, interpretation of findings, and consideration of limitations. For cross-sectional studies, the checklist includes eight items focusing on inclusion criteria, a detailed description of study subjects and setting, valid and reliable measurement of exposures and outcomes, identification of confounding factors, and appropriate statistical analysis. Based on the scoring system, studies were rated as follows: 8–10 = high quality, 5–7 = moderate quality, and ≤4 = low quality. The objective of this appraisal was to evaluate the rigor of all reviewed studies rather than to exclude any full-text articles. The JBI tools consist of checklists tailored for different study designs, including qualitative research, cross-sectional studies, and randomized controlled trials. Additionally, the Mixed Methods Appraisal Tool (MMAT) version 2018 was used to assess the quality of mixed-methods studies (33). The grading of the included studies followed the scoring framework established by (34). Two reviewers (AA and FO) independently conducted the appraisal, with oversight from a senior author (MA, PD) to maintain consistency and objectivity, and resolved discrepancies. The extracted data were analyzed using thematic content analysis, summarized, and synthesized qualitatively. A comprehensive overview of the appraised studies is available in Supplementary Appendix A.

### Data extraction

A structured data extraction process was used to ensure consistency and accuracy in retrieving relevant information from the included studies. A standardized data extraction form was developed and used to collect key details, including author(s), year of publication, country, study design, sample size, population, level of awareness, level of knowledge, and protective and risk factors. Four researchers in two groups independently conducted the data extraction process to minimize errors and biases (AA, FO, YM, BO). Discrepancies were resolved through discussion or consultation with a third reviewer. Extracted data were organized into thematic categories to facilitate synthesis and analysis. The extracted information was further reviewed to ensure completeness and relevance to the study objectives. A summary of the extracted data is presented in Supplementary Appendix 1.

### Data synthesis

A thematic synthesis approach was employed to analyze and integrate findings from the included studies. Extracted data were categorized based on key themes related to preconception awareness, knowledge levels, and influencing factors among women in Africa. A qualitative synthesis was conducted to identify patterns, similarities, and differences across studies, ensuring a comprehensive understanding of the topic. The synthesis process involved comparing results across different study designs to ensure robustness and reliability. Discrepancies in findings were explored and discussed concerning methodological differences and study contexts. The final synthesis was structured to provide an in-depth interpretation of the evidence, highlighting key insights, gaps, and implications for policy and practice.

## Results

### Search results

A total of 52,262 records were identified through database searches across major registers, including PubMed (187), AJOL (83), Web of Science (1,081), Scopus (896), and Dimension AI (50,015). After removing 15,932 duplicate records, 7,968 records marked as ineligible by automation tools, and 1,894 records excluded for other reasons, 26,468 records were screened. Of these, 26,394 were excluded for irrelevance, leaving 74 reports sought for retrieval. Twenty-two reports were not retrieved, and 55 full-text articles were assessed for eligibility. Subsequently, 28 reports were excluded for reasons including non-English language (*n* = 9), studies conducted outside Africa (*n* = 10), and those not reporting variables of interest (*n* = 9). Ultimately, 27 studies met the inclusion criteria and were incorporated into the final systematic review. The PRISMA flowchart is shown in Figure 1.

### Characteristics of included studies

**Figure 2:**
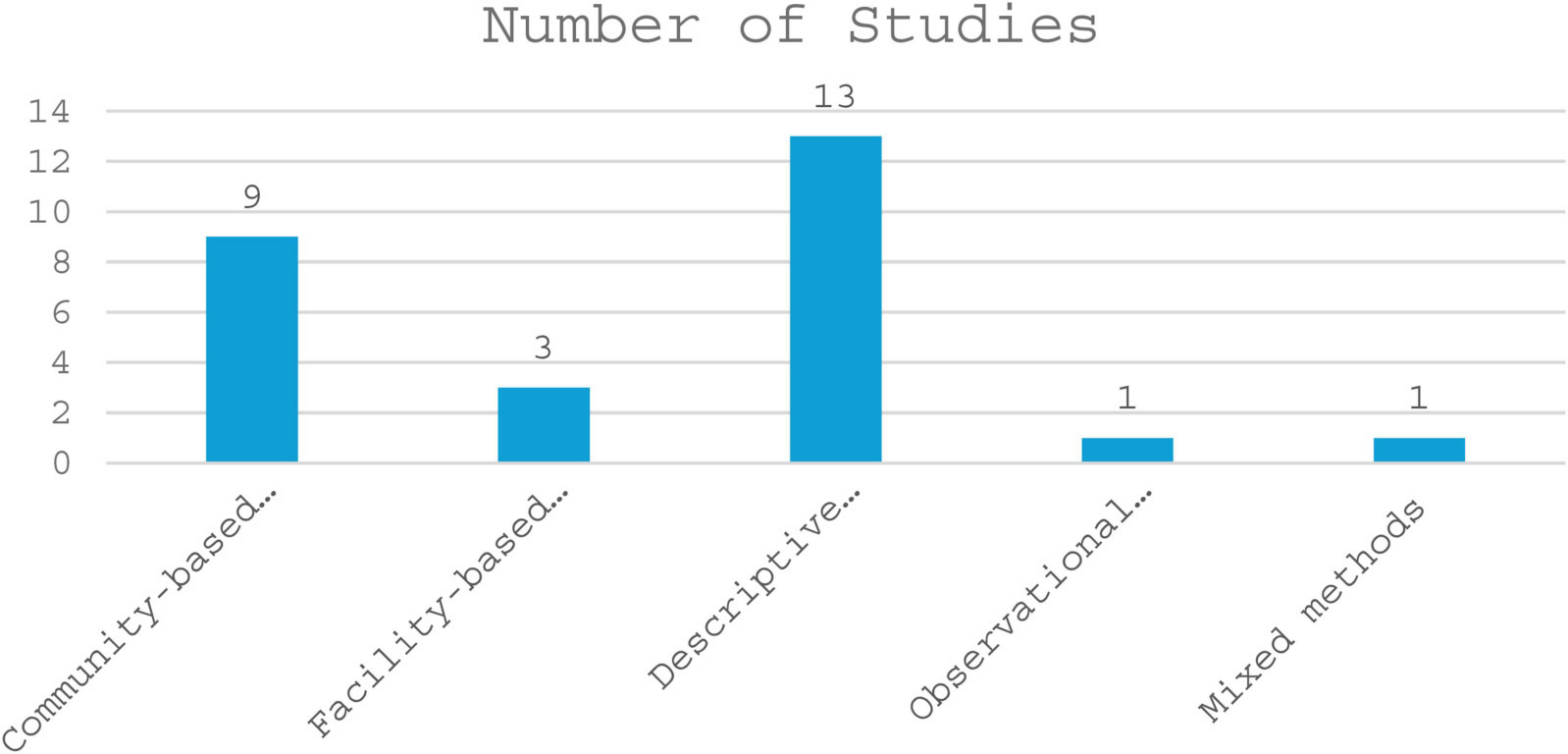
Designs used in the included studies.

**Figure 3:**
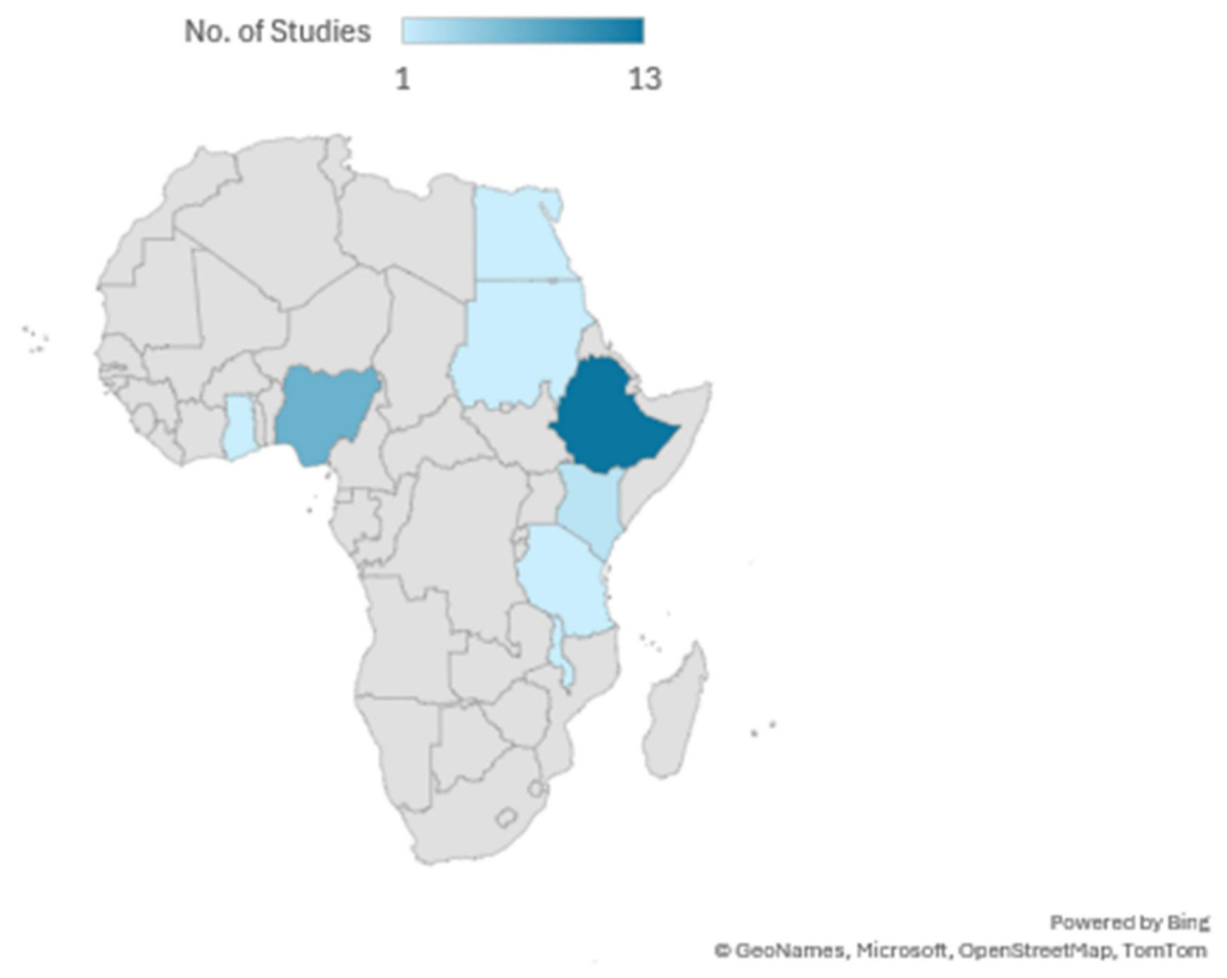
Countries where included studies were conducted.

**Figure 4:**
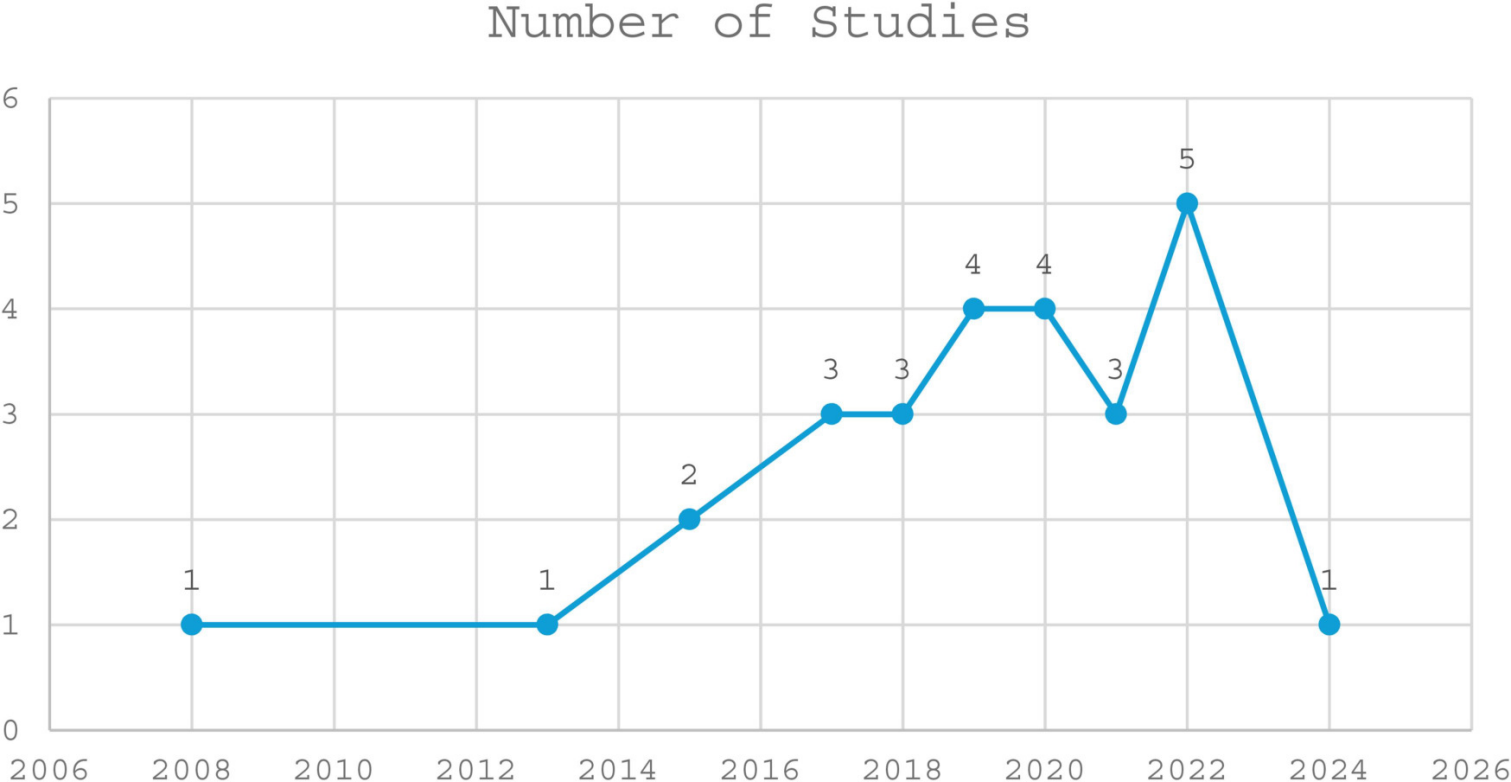
Years of included studies.

**Figure 5:**
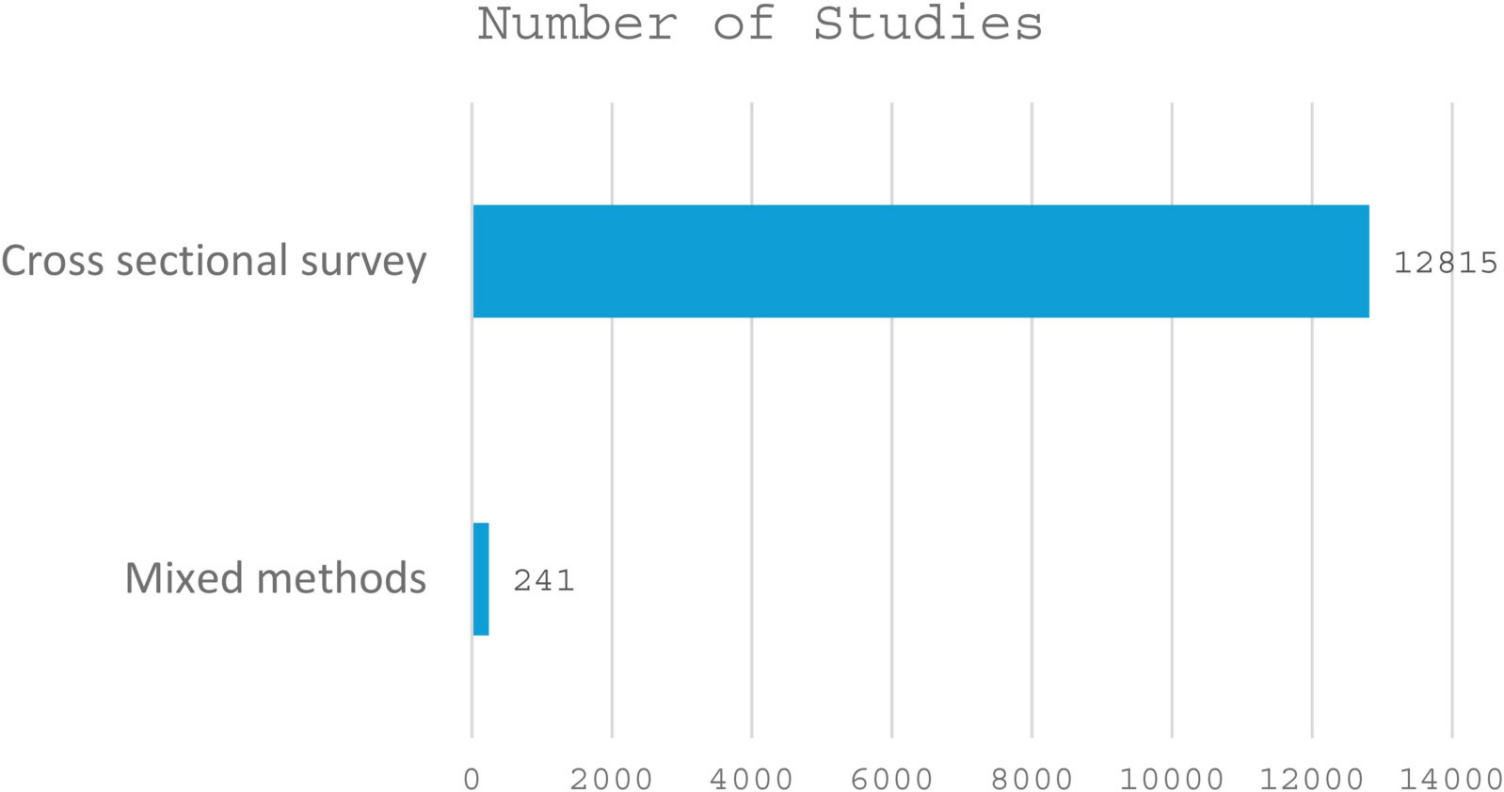
Sample size of included studies (*N* = 13,056).

Out of the 27 included studies, the majority were descriptive cross-sectional studies (13), followed by community-based cross-sectional studies (9) and facility-based cross-sectional studies (3). Observational cross-sectional and mixed-methods studies were the least common, with only one study each (see details in Figure 2). Most of the included studies were conducted in Ethiopia (13), followed by Nigeria (7), Kenya (2), and Sudan (1). Ghana, Egypt, Malawi, and Tanzania had one study each (see details in Figure 3). A total of 27 studies were published between 2008 and 2024, with the highest number of studies published in 2022 (5 studies), followed by 2019 and 2020 (4 studies each), and 2017, 2018, and 2021 (3 studies each) (Figure 4). The earliest studies were published in 2008 and 2013, with only one study each (see details in Figure 5). The total sample size was 12,056.

### Appraisal results

**Figure 6:**
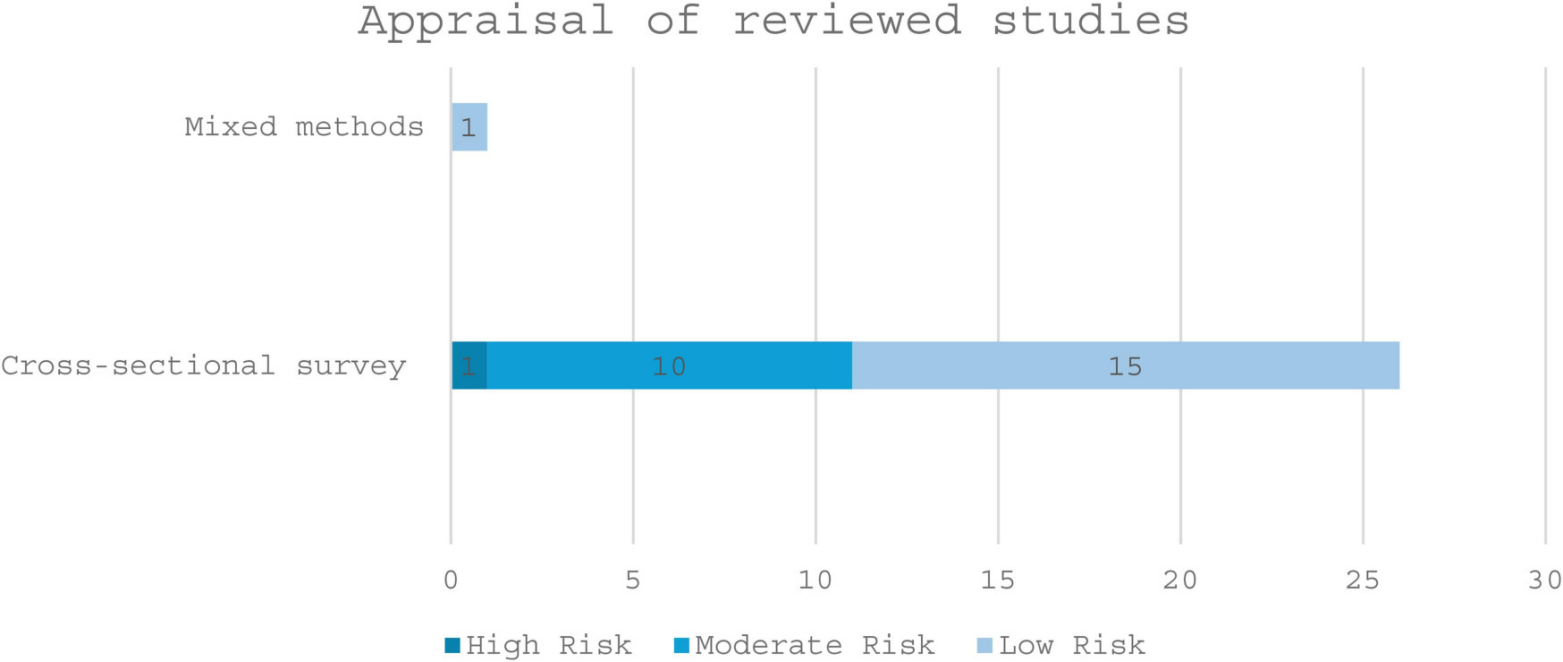
Appraisal of reviewed studies.

The results of appraised studies were categorized into low, moderate, and high-risk. Details are presented in Figure 6. Most notably, confounding factors were not identified in at least seven studies, including Akinajo et al. (46) and Al-Darzi et al. (43). Similarly, no strategies to deal with confounding were stated in even more cases, affecting the credibility of results. A few studies, such as Khonje (52), also lacked valid exposure measurement and standardized outcome criteria. In the MMAT appraisal, Oketch et al. (54) did not address inconsistencies between qualitative and quantitative findings. Still, the majority of studies scored 7 or 8 out of 8, indicating overall strong methodological quality.

### Level of awareness and knowledge of preconception care among women of reproductive age in Africa

#### Level of awareness

Our reviewed studies reported awareness levels ranging from 15.9% in Ethiopia (35) to 91% in Tanzania (36). Few studies documented very low awareness levels, including Kenya (37) at 19% and Nigeria (38) at 20.61%. Several studies indicated moderate awareness levels, such as Nigeria (39) at 42.2%, Nigeria (40) at 43.1%, Nigeria (41) at 59.9%, and Ethiopia (42) at 60.8%. Additionally, higher awareness was observed in Egypt (43) at 62.4%, Ethiopia (44) at 63.2%, and Nigeria (45) at 63.5%. Studies reporting the highest awareness levels included Nigeria (46) at 76%, Nigeria (47) at 87% low awareness, implying 13% high awareness, and Tanzania (36) at 91% (see Table 3).

**Table 3:** Level of awarenes**s.**

Authors, years/country	Purpose	Design	Population	Sample size	Level of awareness
Abrha et al. (48)	To determine the level of women's awareness and associated factors of preconception care service	Cross-sectional survey (community)	Mothers	564	39.0%
Ethiopia					
Aiyejina (47)	To assess knowledge and use of folic acid among female students of reproductive age	Cross-sectional survey	Female students of Reproductive age	418	Low awareness—87%
Nigeria					
Akinajo et al. (46)	To determine the level of awareness, knowledge, and practice of PCC and to identify factors influencing the uptake and utilization of this care among women	Cross-sectional survey	Pregnant women	50	76%
Nigeria					
Boakye-Yiadom et al. (49)	To assess the awareness, knowledge, attitude, and practice of pregnant women attending ANC	Cross-sectional survey	Women aged 16–40 years	200	34.5%
Ghana					
Al-Darzi et al. (43)	To measure the level of knowledge about the periconceptional use of folic acid among pregnant women	Cross-sectional survey (observational)	Pregnant women aged 18–45 years	660	62.4%
Egypt					
Demisse et al. (50)	To assess the preconception care utilization and determine factors that influence the uptake	Cross-sectional survey (community)	Reproductive-age women	410	35.4%
Ethiopia					
Edalia (37)	To determine the level of knowledge on preconception among women at the reproductive health clinic	Cross-sectional survey	Reproductive-age women aged 15–49 years	224	19%
Kenya					
Ekem et al. (39)	To assess the level of awareness and utilisation of PCC services.	Cross-sectional survey	Pregnant women	450	42.2%=aware
Nigeria					
Ezegwui et al. (40)	To determine the awareness and practice of preconception care	Cross-sectional survey	Pregnant women	1,331	43.1%
Nigeria					
Fekene et al. (51)	To identify the level of women's knowledge, uptake, and associated factors of PCC	Cross-sectional survey (community)	Reproductive-age women	669	22.1%
Ethiopia					
Fikadu et al. (44)	To assess knowledge of preconception health, its relation to planned pregnancy, parity, family planning use, and education among married women	Cross-sectional survey (community)	Married women	337	63.2%=aware
Ethiopia					
Goshu et al. (35)	To assess women's awareness of preconception folic acid supplementation and associated factors	Cross-sectional survey (community)	Reproductive-age women	422	15.9% = had good awareness
Ethiopia					
Kachiro et al. (41)	To assess the awareness and perception of preconception care among women	Cross-sectional survey	Women	177	59.9%
Nigeria					
Khonje (52)	To explore the knowledge, attitudes, and practices of pregnant women on preconception care	Cross-sectional survey	Pregnant women	767	24.3%
Malawi					
Lemma et al. (53)	To assess knowledge of PCC and the associated factors among reproductive-age women	Cross-sectional survey (community)	Reproductive-age women	414	35.5%
Ethiopia					
Msigwa (36)	To assess the knowledge and practice of preconception care among women attending reproductive health clinics	Cross-sectional survey	Reproductive-age women aged 17–49 years	424	91%
Tanzania					
Oketch et al. (54)	To identify the factors influencing preconception care services among women of reproductive age	Mixed methods	Reproductive-age women	241	30% = had no idea of the PCC
Kenya					
Olowokere et al. (45)	To determine the level of awareness and knowledge of preconception care	Cross-sectional survey	Women	375	63.5%
Nigeria					
Tesema et al. (42)	To assess knowledge of preconception healthcare and associated factors: a study among mothers	Cross-sectional survey (community)	Reproductive-age women	522	60.8%
Ethiopia					
Umar et al. (38)	To assess awareness and perception of preconception care among women.	Cross-sectional survey	Women	131	20.61%= aware
Nigeria					79.38%= unaware

#### Level of knowledge

Table 4 presents the level of knowledge of the included studies. The reviewed studies reported knowledge levels ranging from 11% in Sudan (55) to 70% in Tanzania (36), with 84% of participants in Malawi having no knowledge (52). Higher knowledge was found in 65.3% in Nigeria (45), 68.6% in Ethiopia (56), 70% in Tanzania (36), and 71.1% in Nigeria (46). However, moderate knowledge was observed in 34% in Ghana (49), 42% in Kenya (37), 44.1% in Nigeria (41), 51.1% in Ethiopia (42), 53% in Ethiopia (57), 55.2% in Ethiopia (44), and 55.6% in Ethiopia (58). Additionally, several studies documented poor knowledge of PCC, including 11% in Sudan (55), 12% in Egypt (43), and 17.1% in Ethiopia (53). Other studies reported 17.3% in Ethiopia (50), 20% in Ethiopia (59), 21.3% in Ethiopia (60), 26.8% in Ethiopia (51), and 31.7% in Nigeria (39). Additionally, 31.8% in Ethiopia (61) and 33.7% in Nigeria (47) had good knowledge, while the remaining had poor knowledge.

**Table 4:** Level of knowledge**.**

Authors, years/country	Purpose	Design	Population	Sample size	Level of knowledge
Ahmed et al. (55)	To study the knowledge, attitude, and practice of preconception care among women with rheumatic heart disease in reproductive age	Cross-sectional survey (facility)	Reproductive-age women aged 15–45 years	100	11%
Sudan					
Aiyejina (47)	To assess knowledge and use of folic acid among female students of reproductive age	Cross-sectional survey	Female students of reproductive age	418	Poor knowledge—66.3%
Nigeria					
					Good knowledge, 33.7%
Akinajo et al. (46)	To determine the level of awareness, knowledge, and practice of PCC and to identify factors influencing the uptake and utilization of this care among women	Cross-sectional survey	Pregnant women	50	71.1%
Nigeria					
Ayalew et al. (61)	To assess women's knowledge and associated factors in preconception care	Cross-sectional survey (community)	Women	422	31.8%
Ethiopia					
Boakye-Yiadom et al. (49)	To assess the awareness, knowledge, attitude, and practice of pregnant women attending ANC	Cross-sectional survey	Women aged 16–40 years	200	42.5% = had poor knowledge
Ghana					34% = moderate knowledge
					23.5% = high knowledge on PCC.
Al-Darzi et al. (43)	To measure the level of knowledge about the periconceptional use of folic acid among pregnant women	Cross-sectional survey (observational)	Pregnant women aged 18–45 years	660	12.0%
Egypt					
Demisse et al. (50)	To assess the preconception care utilization and determine factors that influence the uptake	Cross-sectional survey (community)	Reproductive-age women	410	17.3% = good knowledge
Ethiopia					
Edalia (37)	To determine the level of knowledge on preconception among women at the reproductive health clinic	Cross-sectional survey	Reproductive-age women aged 15–49 years	224	42%
Kenya					
Ekem (39)	To assess the level of awareness and utilization of PCC services.	Cross-sectional survey	Pregnant women	450	31.7% = had good knowledge
Nigeria					
Fekene et al. (51)	To identify the level of women's knowledge, uptake, and associated factors of PCC	Cross-sectional survey (community)	Reproductive-age women	669	26.8% = had a good knowledge
Ethiopia					
					73.2% = had inadequate knowledge
Fikadu et al. (44)	To assess knowledge of preconception health, its relation to planned pregnancy, parity, family planning use, and education among married women	Cross-sectional survey (community)	Married women	337	55.2%
Ethiopia					
Gamshe and Demissie (56)	To identify perinatal factors affecting knowledge and utilization of preconception care among pregnant women	Cross-sectional survey	Pregnant women	331	68.6% = have good knowledge
Ethiopia					
					31.4% = had poor knowledge
Kachiro et al. (41)	To assess the awareness and perception of preconception care among women	Cross-sectional survey	Women	177	44.1%
Nigeria					
Kassa and Yohannes (59)	To assess the level of knowledge and associated factors toward preconception care among mothers who gave birth	Cross-sectional survey (Facility)	Pregnant women	580	20% = had good knowledge
Ethiopia					
					80% = poor knowledge
Khonje et al.(52)	To explore the knowledge, attitudes, and practices of pregnant women on preconception care	Cross-sectional survey	Pregnant women	767	84% = no idea about PCC
Malawi					
Demeke et al. (58)	To assess knowledge and attitude toward preconception care and associated factors among women of reproductive age with chronic disease	Cross-sectional survey	Reproductive-age women	844	55.6% = had good knowledge
Ethiopia					
Lemma et al. (53)	To assess knowledge of PCC and the associated factors among reproductive-age women	Cross-sectional survey (community)	Reproductive-age women	414	17.1% = had good knowledge of PCC
Ethiopia					
					82.9% = poor knowledge
Msigwa (36)	To assess the knowledge and practice of preconception care among women attending reproductive health clinics	Cross-sectional survey	Reproductive-age women aged 17–49 years	424	70% had good knowledge of PCC
Tanzania					
Olowokere et al. (45)	To determine the level of awareness and knowledge of preconception care	Cross-sectional survey	Women	375	65.3% = good knowledge
Nigeria					
					34.1% = poor knowledge
Tesema et al. (42)	To assess knowledge of preconception healthcare and associated factors: a study among mothers	Cross-sectional survey (community)	Reproductive-age women	522	51.1%.
Ethiopia					
Teshome et al. (60)	to assess the level of knowledge of PCC and associated factors among pregnant women	Cross-sectional survey (community)	Pregnant women	623	21.3% = had good knowledge of PCC.
Ethiopia					
Yohannes et al. (57)	To assess the levels and correlates of knowledge and attitude of preconception care among mothers who gave birth at public hospitals	Cross-sectional survey (facility)	Women	370	53% = had good knowledge
Ethiopia					

### Factors influencing the awareness and knowledge of preconception care among women in Africa

#### Risk factors

Risk factors influencing maternal and reproductive health outcomes are shaped by a wide range of sociodemographic, economic, obstetric, reproductive, maternal health, and systemic health service conditions. Sociodemographic variables such as young age (36, 39, 41, 48, 54), young-to-middle adulthood (61), and marital status (54) or unemployment (38) contribute significantly to increased vulnerability. Educational attainment presents a dual pattern: Individuals with tertiary education (39–41, 45, 49) as well as those with primary or secondary education and above (35, 42, 44, 51, 54, 57, 58, 61) have been associated with diverse risk levels. The educational background of spouses, particularly secondary education or higher, also appears to influence PCC awareness (59, 60). Additionally, ethnic and religious identity such as being Hausa (38) or identifying as Muslim (49) and place of residence, especially urban environments (39, 59), affect maternal health access and outcomes. Professional roles also intersect with risk factors, which include civil servants (41), non-governmental organization (NGO) workers (51), market vendors (44), daily laborers (53), housewives (42), and self-employed individuals (54), all face unique health challenges.

Additionally, economic conditions significantly contribute to maternal health risk. Women in the middle-income bracket often fall into service gaps that worsen outcomes (35, 48, 53, 59), while a lack of financial support compounds access issues (36). Obstetric history remains one of the strongest predictors of maternal risk. Gravidity, whether ≤2 (43) or >2 (53), and parity, including parity of 1 (58), between 2 and 3 (41), or >2 (44), all appear as significant indicators. Prior adverse pregnancy experiences, such as stillbirth or neonatal death (42, 53), miscarriage (55), congenital abnormalities (53), or complications such as pregnancy-induced hypertension (59), further elevate the risk. Chronic illnesses (60), perinatal complications (56), macrosomia in previous infants (59), and long-term disease durations (58) underscore the need for tailored clinical attention. Moreover, reproductive health behaviors play a key role. Past or current use of family planning services (35, 42, 44, 48, 51, 53, 55, 61), contraceptive history (53, 55), and the intention behind pregnancy, whether planned or not, shape health trajectories (44, 60). Additionally, belief in the benefits of preconception care and receiving counseling against pregnancy serve as moderating influences (55). Service utilization among women, such as attending at least one antenatal care (ANC) session (59), delivering at a health facility (39), or receiving postnatal care (PNC) (51, 56), is shown to improve outcomes while also often being prompted by high-risk status. In some cases, early diagnosis of chronic health problems, such as during childhood or pregnancy, further necessitates intensive monitoring (55). Moreover, health system-related factors compound these risks. Long distances to health facilities, particularly walking 34 min or more, pose physical and logistical challenges (53). Although institutional deliveries are often beneficial, they may also reflect underlying complications prompting facility referrals (51) (see Table 5).

**Table 5:** Risk factors influence the awareness and knowledge of preconception care among women in Africa.

Theme	Specific risk factor	Authors
Sociodemographic factors	Young adult	(36, 39, 41, 48, 54)
	Young and middle-aged adults	(61)
	Having a tertiary education	(39–41, 45, 49)
	Having a primary, secondary education and above	(35, 42, 44, 51, 54, 57, 58, 61)
	Spouse having secondary education and above	(59, 60)
	Being in primary school	(55)
	Being a Hausa	(38)
	Being a Muslim	(49)
	Living in an urban area	(39, 59)
	Being a civil servant	(41)
	Working as an NGO employee	(51)
	Being a market trade vendor	(44)
	Working as a daily laborer	(53)
	Being a housewife	(42)
	Married	(54)
	Being a self-employed	(54)
	Being unemployed	(38)
Economic factors	Middle income	(35, 48, 53, 59)
	Not having financial support.	(36)
Obstetric factors	Gravidity ≤2	(43)
	Gravidity > 2	(53)
	Parity between 2 and 3	(41)
	Parity > 2	(44)
	Parity (1)	(58)
	Previous adverse pregnancy outcome	(48)
	Having a history of neonatal death	(42, 53)
	Miscarriages	(55)
	Having a history of congenital abnormality	(53)
	Having a lesion	(55)
	Having a history of Pregnancy-Induced Hypertension	(59)
	Having a pre-existing illness	(60)
	Impact of Rheumatic Heart Disease (RHD) on pregnancy	(55)
	Having a duration of disease ≥ 5 years	(58)
	Having a history of perinatal complications	(56)
	Having a history previous infant with macrosomia	(59)
	Having a health problem	(35)
Reproductive health factors	Previous/History of family planning use	(35, 42, 44, 48, 51, 61)
	Uses of FP	(55)
	Having a history of contraceptive use	(53)
	Planned pregnancy	(44, 60)
	Believe in benefit of PCC	(55)
	Being counseled against pregnancy	(55)
Maternal health factors	Having attended at least one ANC visit	(59)
	Delivery in a health facility	(39)
	Attended PNC for the previous delivery	(56)
	Utilized PNC service	(51)
	Childhood and pregnancy as a time of diagnosis	(55)
Health system factors	Time to reach health facility ≥34 min (on foot)	(53)
	Having delivered in a health institution	(51)

#### Protective factors

Several studies identified a range of protective factors associated with improved maternal health outcomes and behaviors. Protective factors that shape women's awareness and knowledge of PCC in African contexts span a range of individual, reproductive, economic, and systemic dimensions. Age and education are significant, with increased awareness consistently linked to being a teenager or young adult (38, 43, 45, 46, 49, 55, 60), having a tertiary or secondary education (43, 46), being enrolled in primary or secondary education (36), or simply having some level of formal schooling (38). Interestingly, a few studies also report greater awareness among women with no formal education (48, 57). Ethnicity and marital status also matter, and being Yoruba or Hausa (41, 46) and being married (36, 39, 46, 49) have been positively associated with PCC awareness. Urban residency further enhances information access and proximity to services (60).

Additionally, reproductive history strengthens awareness, where previous pregnancies (parity) and gravidity levels between one and four are linked with higher knowledge (38, 39, 46, 48, 55, 58). Additionally, the absence of adverse reproductive outcomes such as stillbirths, preterm births, and abortions correlates with greater awareness (41). Women who reported having planned their pregnancies also showed better understanding of PCC services (57). Again, socioeconomic status contributes to awareness in nuanced ways. Women working as civil servants, students, government employees, housewives, or business owners report greater awareness (42, 46, 51, 53, 57). Surprisingly, unemployment is not always a barrier, as some unemployed women still exhibited high awareness likely due to informal education or targeted public health interventions (36, 43). Monthly income levels between 1001 and 5,000 birr also appear to be protective, possibly enabling better access to health education and services (53, 59).

Health service access and engagement are among the strongest determinants of PCC awareness. Women who had adequate ANC during pregnancy (43), used long-acting family planning methods prior to conception (60), or received counseling on PCC (58), health education (36), and targeted information during the perinatal period (56) were significantly more informed. In addition, medical and surgical history also shaped knowledge. Women with no chronic diseases (48), no history of neonatal death (42), or those who had corrective surgery (55) demonstrated better awareness, possibly due to improved health status or closer engagement with the health system. Social support is a key facilitator. Women who discussed reproductive plans jointly with their partners or received support from husbands were more likely to seek information and understand PCC services (48). Finally, access to information sources greatly influenced awareness levels. Health professionals such as midwives and doctors, along with media exposure through radio and television, played key roles (36, 57, 60). Community meetings and interpersonal networks, including healthcare provider friends or relatives, also served as powerful conduits for disseminating PCC knowledge (57) (see Table 6).

**Table 6:** Protective factors influence the awareness and knowledge of preconception care among women in Africa.

Theme	Specific protective factor	Authors
Sociodemographic factors	Being a young adult	(38, 43, 45, 46, 49, 55)
	Being a teenager and young adult	(60)
	Having no education	(48, 57)
	Having a tertiary education	(46)
	Secondary education	(43)
	Being in primary and secondary education	(36)
	Having a formal education	(38)
	Being a Yoruba or Hausa	(41, 46)
	Being married	(36, 39, 46, 49)
	Living in urban area	(60)
Reproductive health factors	Parity	(38, 39, 46, 48, 55, 58)
	Gravidity 1 and 2–4	(48)
	Stillbirths ≤ 0	(41)
	Preterm births ≤ 0	(41)
	Abortions ≤ 0	(41)
	Had pregnancy planned	(57)
Socioeconomic factors	Being a civil/government servant,	(46, 51)
	Being a housewife, student, government employee, and private business	(42, 51, 53)
	Not employed	(36, 43)
	Monthly income between 1,001–5,000 birr	(53, 59)
Health services and access factors	Sufficient ANC visits in the current pregnancy	(43)
	Using a long-acting family planning prior to the recent pregnancy	(60)
	Received health education	(36)
	Receiving PCC counseling	(58)
	Being informed about PCC in perinatal periods	(56)
Medical and surgical history	Not having any chronic diseases	(48)
	Had corrective surgery	(55)
	No history of neonatal death	(42)
Social support system	Having joint plan discussion with partner	(48)
	Having husband support	(48)
Health information sources	Midwives, doctors' and media as sources	(36)
	Community meetings related to PCC	(57)
	Had a radio/TV	(57, 60)
	Having healthcare provider friends	(57)
	Having healthcare provider relatives	(57)

## Discussion

### Summary of findings

Evidence shows that many individuals have low awareness and knowledge about PCC, with awareness levels ranging from 15.9% to 91% and knowledge levels varying from poor (as low as 11%) to good (up to 71.1%). Risk factors associated with low awareness and poor knowledge include being unemployed, having no formal education, residing in rural areas, and belonging to marginalized ethnic or religious groups, such as the Hausa or Muslim populations in certain settings. Socioeconomic vulnerability, including low or unstable income and lack of financial support, further impedes access to information and services. Obstetric and reproductive factors such as high parity, gravidity beyond two, a history of adverse pregnancy outcomes, and pregnancy-related complications were also linked to reduced PCC engagement. Additionally, women with chronic illnesses, prolonged disease duration, or who had never used family planning services demonstrated poorer knowledge. Conversely, protective factors included higher maternal education, being a young or middle-aged adult, urban residence, and being employed in formal sectors such as civil service or NGOs. Reproductive characteristics such as planned pregnancies, moderate gravidity and parity, and the absence of stillbirths or abortions promoted timely healthcare-seeking behaviors. Adequate antenatal and postnatal care attendance, institutional delivery, prior use of long-acting family planning, receipt of preconception counseling, and access to health education via media or healthcare providers further strengthened maternal preparedness. Supportive environments involving partner support, joint decision-making, community engagement, and social networks with health-informed individuals also served as critical enablers of positive PCC behaviors. Interventions aimed at addressing these modifiable risk and protective factors are vital to improving PCC utilization and maternal health outcomes across diverse populations.

### Level of awareness and knowledge of preconception care among women of reproductive age in Africa

The observed variations in awareness levels regarding PCC highlight disparities influenced by healthcare accessibility, education, and public health initiatives. Extremely low awareness levels in Ethiopia (5.9%) (35), Kenya (19%) (37), and Nigeria (20.61%) (38) suggest significant gaps in public health education, possibly due to limited outreach programs and inadequate dissemination of reproductive health information. Moderate awareness levels in Nigeria, including 42.2% (39), 43.1% (40), and 59.9% (41), as well as in Ethiopia (60.8%) (42), indicate some progress, but the figures suggest a need for intensified awareness campaigns to reach broader populations. Higher awareness levels in Egypt (62.4%) (43), Ethiopia (63.2%) (44), and Nigeria (63.5%) (45) suggest that structured interventions, including health education and media campaigns, contribute positively to knowledge dissemination. The highest awareness levels observed in Nigeria, including 76% (46) and 87% (47), as well as Tanzania (91%) (36), may reflect stronger healthcare systems, better educational outreach, or increased engagement with maternal health programs. These findings emphasize the need for targeted interventions to address regional disparities and ensure equitable access to PCC awareness through policy-driven educational programs and community-based initiatives.

Similarly, terms of knowledge levels regarding PCC reflect disparities in healthcare access, education, and public health interventions across different regions. Extremely low knowledge levels in Sudan (11%) (55) and Egypt (12%) (43) may be attributed to inadequate health education programs and limited awareness campaigns targeting reproductive health. Similarly, poor knowledge in Ethiopia, ranging from 17.1% to 31.8% (53, 61), suggests potential barriers such as low literacy levels, cultural misconceptions, and inadequate integration of PCC into routine healthcare services. Moderate knowledge levels in Ghana (34%) (49), Kenya (42%) (37), and Nigeria (44.1%) (41) indicate some progress but highlight the need for enhanced educational interventions to bridge existing gaps. Higher knowledge levels in Nigeria (65.3%) (45), Ethiopia (68.6%) (56), and Tanzania (70%) (36) suggest that structured awareness campaigns and improved healthcare accessibility contribute to increased PCC understanding. Despite these positive trends, the persistence of knowledge gaps underscores the necessity for targeted public health strategies, including community-based education, healthcare provider training, and policy-driven interventions to ensure equitable access to PCC information and services.

### Factors influencing the awareness and knowledge of preconception care among women in Africa

Maternal health-seeking behavior and outcomes are shaped by a range of interconnected risk factors spanning sociodemographic, economic, obstetric, reproductive, maternal health, and systemic domains. These factors operate at individual, household, and structural levels, influencing both access to and utilization of maternal and reproductive health services. Sociodemographic factors are particularly significant in shaping women's health behaviors and vulnerabilities. Younger women, especially those in early adulthood, are more likely to experience poor maternal outcomes due to limited reproductive experience, lower autonomy, and inadequate access to resources (36, 41, 48). Age thus intersects with decision-making capacity and social support. Similarly, educational attainment, whether by the woman or her spouse, emerges as a crucial predictor. Women with secondary education and above generally demonstrate improved maternal health literacy, which translates into better uptake of ANC, family planning, and facility-based deliveries (35, 42, 44). Conversely, some studies associate even tertiary education with risk, possibly reflecting heightened reporting, awareness, or expectations from the health system (45, 49). Occupational status also influences risk. Civil servants, NGO workers, housewives, market traders, and daily laborers each face different levels of exposure to health information and healthcare access, depending on the demands of their roles and flexibility of time (51, 53). Religious and ethnic identity, such as being Muslim or Hausa, has been linked to maternal health outcomes, suggesting that cultural or faith-based norms may either restrict or enable access to reproductive services (38, 49). Urban residency, while often associated with better access, may also present unique barriers, including overcrowding or under-resourced facilities (39, 59).

Economic factors further compound health vulnerabilities. Middle-income women, paradoxically, may be underserved if they do not qualify for subsidies yet cannot afford private care, creating a “missing middle” in health equity (48, 53). Lack of financial support significantly reduces a woman's ability to seek timely care, attend checkups, or purchase medication, highlighting the persistent role of poverty in maternal health disparities (36). Additionally, obstetric factors, such as gravidity, parity, and prior adverse pregnancy outcomes, were consistently identified across studies. Both low (primigravida) and high parity levels were associated with increased risks, although the direction of risk varied depending on whether the concern was for complications from inexperience or from cumulative health strain (43, 44). Prior negative experiences such as neonatal deaths, miscarriages, congenital abnormalities, or pregnancy-induced hypertension indicate higher vulnerability in subsequent pregnancies (53, 59). Chronic illness, disease duration of >5years, and history of macrosomia also predict adverse outcomes, underscoring the importance of detailed obstetric histories during clinical assessments (56, 58). Again, reproductive health-related behaviors, such as contraceptive use, family planning history, and pregnancy intentionality, are also influential. Women who previously used contraceptives or accessed family planning services may have varied outcomes depending on the type, method, and continuity of care (42, 51). Planned pregnancies are typically associated with better maternal outcomes, yet in some contexts, women with chronic conditions may plan pregnancies under higher risk (60). The belief in preconception care and counseling further reflects the role of proactive health-seeking behaviors in mitigating risk (55). Additionally, maternal health service utilization, such as ANC, postnatal care, and institutional delivery, reflects both awareness and access. While attending at least one ANC visit or utilizing PNC services is generally beneficial, some studies noted associations with risk, likely due to late initiation of care or poor service quality (51, 56). Diagnosis of chronic illness during pregnancy or childhood affects maternal preparedness and outcomes. Finally, health system factors such as distance to facilities and travel time significantly affect women's ability to obtain timely care. Walking >30 min to a facility was associated with reduced access, highlighting how geographic barriers, especially in rural settings, can delay or deter service use (53). Even among women who deliver in institutions, inadequate care or referral delays may undermine the protective role of facility-based childbirth (51).

This review highlights a wide range of protective factors that positively influence maternal and reproductive health outcomes across multiple domains. Sociodemographic characteristics play a central role in shaping women's health behaviors and access to care. Age was a significant determinant, with studies showing that being a young adult or teenager often correlated with increased access to maternal services and better outcomes, likely due to targeted health messaging and educational exposure among younger populations (38, 43, 45, 46, 49, 55, 60). Educational status also had nuanced effects. While tertiary and secondary education were associated with improved health literacy and service uptake (36, 38, 43, 46), some studies also noted protective outcomes among women with no formal education, possibly due to adherence to traditional health practices or strong family and community support systems (48, 57).

Ethnic background and marital status emerged as further protective sociodemographic factors. Belonging to the Yoruba or Hausa ethnic groups, who may benefit from stronger cultural cohesion or supportive kinship structures, was associated with improved maternal outcomes (41, 46). Being married offered emotional and financial support and often led to shared reproductive health decision-making (36, 39, 46, 49). Urban residence also enhanced access to maternal health services due to proximity to better healthcare infrastructure and information dissemination (60). Again, reproductive health history contributed significantly to maternal health protection. Higher parity and moderate gravidity (1–4 previous pregnancies) improved reproductive knowledge and responsiveness to danger signs (38, 46, 48, 58). Additionally, the absence of adverse reproductive outcomes such as stillbirths, preterm births, and abortions was associated with continued confidence in and use of health services (41). Planned pregnancies also contributed to better maternal outcomes, indicating the importance of intentional conception and readiness for parenthood (57). Additionally, socioeconomic factors further influenced maternal health behavior. Formal employment, especially as a civil servant or business owner, increased women's financial autonomy and access to services (42, 46, 51, 53). Interestingly, even being unemployed or a housewife was cited as protective in certain contexts, possibly due to increased availability of time for health facility visits or strong domestic support (36, 43). Moderate household income levels also supported maternal care-seeking behavior by reducing economic barriers to service access (30, 53).

Moreover, access to health services emerged as a powerful protective factor. Consistent ANC attendance ensured timely health monitoring, education, and counseling (43). Prior use of long-acting contraceptives and exposure to PCC counseling were both associated with informed reproductive decisions and better birth preparedness (58, 60). Additionally, being informed about PCC during the perinatal period increased early engagement with maternal health services (56). In terms of medical history, women with no chronic diseases and those who had undergone corrective surgery experienced fewer complications during pregnancy (48, 55). The absence of prior neonatal death also contributed to a stronger trust in healthcare systems and better service utilization (42). Social support systems were strongly associated with positive maternal health behaviors. Joint planning with partners and husband support enhanced emotional security and encouraged early and sustained care-seeking (48). This highlights the importance of male involvement and partner communication in reproductive health. Finally, health information sources such as media, healthcare providers, and community outreach played a crucial role in improving maternal knowledge and behaviors. Exposure to radio and television programs, health-related community meetings, and information from healthcare provider friends or relatives helped build awareness and reinforce positive practices (36, 57, 60). These informal information pathways were especially valuable in settings where formal education or healthcare access was limited.

## Limitations of the review

The scope of this review was limited to studies published in English, which may have influenced the number and diversity of included studies. The authors have analyzed PCC awareness and knowledge across various populations, with a notable concentration of studies from Ethiopia and Nigeria. It is crucial to interpret these findings with caution, as the overrepresentation of specific regions may not accurately reflect global trends in PCC awareness. While the authors appraised the methodological quality of the included studies, the quality did not determine their inclusion in this review. The appraisal revealed that many studies relied on self-reported data, making them susceptible to recall bias, social desirability bias, and inconsistencies in PCC assessment tools. Furthermore, cross-sectional designs were predominant, limiting the ability to establish causal relationships between risk factors and awareness levels. The inclusion of studies with varying methodological rigor may introduce bias into the synthesis of evidence, potentially affecting the reliability of conclusions. Findings from studies with small or non-representative samples may not be generalizable to broader populations. However, the authors approached these limitations with transparency, ensuring that lower-quality studies were carefully interpreted while emphasizing more robust evidence.

### Implications for policy, practice, and research

The findings of this review indicate the urgent need for policymakers to formally integrate PCC into national reproductive and maternal health strategies across African countries. Despite evidence of its role in reducing adverse maternal and neonatal outcomes, PCC remains marginalized in many health systems, particularly in low-resource settings. Governments must prioritize the development of comprehensive PCC policies that extend beyond urban tertiary facilities to encompass community and primary-level health systems. This includes mandating PCC counseling as part of routine family planning, antenatal, and postnatal care, and allocating dedicated funding to support training, public education, and service delivery. Policies should also address structural barriers by subsidizing access to PCC services for economically disadvantaged groups and embedding culturally appropriate communication strategies to reach marginalized populations, including adolescent girls and women with chronic illnesses.

Furthermore, this review highlights a pressing need to enhance provider capacity and service delivery frameworks to ensure PCC is implemented consistently and equitably. Frontline health workers, including midwives, nurses, and community health personnel, must be trained not only in the technical aspects of PCC but also in patient-centered communication, cultural competence, and risk-based screening. Integrating PCC into ongoing community outreach programs and maternal health campaigns can bridge the knowledge gap, particularly in rural areas where awareness remains critically low. Given the influence of social and relational factors, such as spousal support and peer influence, healthcare systems must adopt a more inclusive model that engages men, families, and communities in the preconception health conversation. Additionally, leveraging mass media and digital platforms to disseminate targeted PCC messages can amplify reach, especially among younger, digitally connected populations.

Future research should prioritize understanding the contextual dynamics shaping PCC awareness and uptake across different sub-populations in Africa. While this review mapped common risk and protective factors, more nuanced, mixed-methods studies are needed to unpack how intersecting social determinants, including gender norms, health literacy, digital access, and religious beliefs, mediate knowledge and service utilization. There is also a critical need to develop and validate standardized PCC measurement tools tailored to low-resource contexts, enabling more accurate cross-country comparisons and monitoring. Further, longitudinal and implementation science research should be employed to evaluate the impact of PCC policies and interventions over time, particularly those that are community-driven or technology-enabled. Such evidence will be pivotal in informing scalable, sustainable solutions to improve maternal and newborn health outcomes through early intervention.

## Conclusion

Disparities in PCC awareness and knowledge persist across various populations, influenced by a complex interplay of risk and protective factors. Limited education, low socioeconomic status, rural residence, and restricted healthcare access are key barriers that contribute to inadequate PCC awareness. Cultural beliefs, low male partner involvement, and minimal exposure to health information further hinder knowledge and utilization. In contrast, higher education, stable employment, urban residence, and frequent ANC visits significantly enhance PCC awareness. Media exposure, community engagement, and access to family planning services also play a crucial role in improving knowledge levels. However, challenges remain in addressing these disparities, particularly in resource-limited settings, where weak healthcare infrastructures and socioeconomic inequalities exacerbate gaps in PCC awareness. Strengthening health education initiatives, expanding healthcare access, integrating PCC into routine maternal care services, and leveraging digital platforms for health information dissemination are essential steps toward improving knowledge and utilization. These strategies align with global health priorities to enhance maternal and neonatal health outcomes, ultimately reducing preventable pregnancy-related complications and fostering a more equitable approach to reproductive healthcare.

## Data Availability

The original contributions presented in the study are included in the article/Supplementary Material; further inquiries can be directed to the corresponding author.
