# Supplementary material for: Factors influencing preconception care awareness and knowledge among women in Africa: a systematic review
Source: Front Reprod Health. 2026 Jan 9;7:1702378. doi: 10.3389/frph.2025.1702378 (PMC12827621; doi:10.3389/frph.2025.1702378)
Supplement: Supplementary file 1 [file Table1.docx]

**Appendix B: Appraisal of studies using the JBI and Mixed-method appraisal (MMAT) tools**

**Cross-sectional surveys (JBI)**

| **References** | **Were the criteria for inclusion in the sample clearly defined?** | **Were the study subjects and the setting described in detail?** | **Was the exposure measured in a valid and reliable way?** | **Were objective, standard criteria used for measurement of the condition?** | **Were confounding factors identified?** | **Were strategies to deal with confounding factors stated?** | **Were the outcomes measured in a valid and reliable way?** | **Was appropriate statistical analysis used?** | **Scores** | **Overall Quality** |
| --- | --- | --- | --- | --- | --- | --- | --- | --- | --- | --- |
| Abrha et al. (2020) | Yes | Yes | Yes | Yes | Yes | Yes | Yes | Yes | 8/8 | High |
| Ahhmed et al. (2015) | Yes | Yes | Yes | Yes | No | No | Yes | Yes | 6/8 | Moderate |
| Aiyejina (2017) | Yes | Yes | No | Yes | Yes | No | Yes | Yes | 6/8 | Moderate |
| Akinajo et al. (2019) | Yes | Yes | Yes | Yes | No | No | Yes | Yes | 6/8 | Moderate |
| Ayalew et al. (2017) | Yes | Yes | Yes | Yes | Yes | Yes | Yes | Yes | 8/8 | High |
| Boakye-Yiadom et al. (2020) | Yes | Yes | Yes | Yes | No | Yes | Yes | Yes | 7/8 | High |
| Darzi et al. (2013) | Yes | Yes | Yes | Yes | No | No | Yes | Yes | 7/8 | Moderate |
| Demeke et al. (2024) | Yes | Yes | Yes | Yes | Yes | Yes | Yes | Yes | 8/8 | High |
| Demissie et al. (2019) | Yes | Yes | Yes | Yes | Yes | No | Yes | Yes | 7/8 | High |
| Edalia (2022) | Yes | Yes | Yes | Yes | No | Yes | Yes | Yes | 7/8 | High |
| Ekem (2018) | Yes | Yes | Yes | Yes | Yes | Yes | Yes | Yes | 8/8 | High |
| Ezegwui et al. (2008) | Yes | Yes | Yes | Yes | No | Yes | Yes | Yes | 7/8 | High |
| Fikadu et al. (2022) | Yes | Yes | Yes | Yes | Yes | Yes | Yes | Yes | 8/8 | High |
| Gamshe & Demissie (2022) | Yes | Yes | Yes | Yes | Yes | No | Yes | Yes | 7/8 | High |
| Kachiro et al. (2022) | Yes | Yes | Yes | Yes | Yes | Yes | Yes | Yes | 8/8 | High |
| Fekene et al. (2020) | Yes | Yes | Yes | Yes | Yes | Yes | Yes | Yes | 8/8 | High |
| Goshu et al. (2018) | Yes | Yes | Yes | No | Yes | No | Yes | Yes | 6/8 | Moderate |
| Kassa & Yohannes (2018) | Yes | Yes | No | No | Yes | Yes | Yes | Yes | 6/8 | Moderate |
| Khonje (2017) | Yes | Yes | No | No | No | No | Yes | Yes | 4/8 | Low |
| Lemma et al. (2022) | Yes | Yes | Yes | Yes | Yes | Yes | Yes | Yes | 8/8 | High |
| Msigwa (2021) | Yes | Yes | Yes | No | Yes | No | Yes | Yes | 6/8 | Moderate |
| Olowokere & Owofadeju (2015) | Yes | Yes | No | No | Yes | Yes | Yes | Yes | 6/8 | Moderate |
| Tesema et al. (2021) | Yes | Yes | Yes | Yes | Yes | Yes | Yes | Yes | 8/8 | High |
| Teshome et al. (2020) | Yes | Yes | Yes | Yes | Yes | Yes | Yes | Yes | 8/8 | High |
| Umar et al. (2019) | Yes | Yes | Yes | No | Yes | No | Yes | Yes | 6/8 | Moderate |
| Yohanees et al. (2019) | Yes | Yes | Yes | No | Yes | Yes | Yes | Yes | 7/8 | High |

**Mixed method (MMAT)**

| **References** | **Is there an adequate rationale for using a mixed methods design to address the research question?** | **Are the different components of the study effectively integrated to answer the research question?** | **Are the outputs of the integration of qualitative and quantitative components adequately interpreted?** | **Are divergences and inconsistencies between quantitative and qualitative results adequately addressed?** | **Do the different components of the study adhere to the quality criteria of each tradition of the methods involved?** | **Scores** | **Overall Quality** |
| --- | --- | --- | --- | --- | --- | --- | --- |
| **Oketch et al. (2021)**​ | Yes | Yes | Yes | No | Yes | 4/5 | High |
