# Supplementary material for: Factors influencing preconception care awareness and knowledge among women in Africa: a systematic review
Source: Front Reprod Health. 2026 Jan 9;7:1702378. doi: 10.3389/frph.2025.1702378 (PMC12827621; doi:10.3389/frph.2025.1702378)
Supplement: Supplementary file 3 [file Table3.docx]

**Appendix 1: Extracted Data**

| **Authors, years/country** | **Purpose** | **Design** | **Population** | **Sample size** | **Level of awareness** | **Level of knowledge** | **Risk factors** | **Protective factors** |
| --- | --- | --- | --- | --- | --- | --- | --- | --- |
| Abrha et al 2020  Ethiopia | To determine the level of women’s  awareness and associated factors of preconception-care  service | Community based cross-sectional study | mothers | 564 | 39.0% |  | Young adult  Household income in ETB [501–1000]  Previous family planning use  Previous adverse pregnancy outcome  Having joint plan discussion with partner  Having husband support | Mother’s education (no education and above)  Parity ≤1  Gravidity 1 and 2-4  Not having any chronic diseases |
| Ahmed et al 2015  Sudan | To study knowledge, attitude  and practice of preconception care among women  with rheumatic heart disease in reproductive age | Facility based cross-sectional study | Reproductive women  age (15- 45 years) | 100 |  | 11% | Believe in benefit of preconception care  Childhood and pregnancy as a time of diagnosis  Impact of RHD on pregnancy  Miscarriages  Having a lesion  Uses of FP  Being counselled against pregnancy  Being in primary school | Had corrective surgery  Parity ≥ 2  Young adults |
| Aiyejina, 2017  Nigeria | To assess knowledge and use of folic acid among female students of reproductive age | Descriptive cross-sectional study | Female students of reproductive age | 418 | Low awareness—87% | Poor knowledge—66.3%  Good knowledge, 33.7% | Marital status  Study level | Economic status |
| Akinajo et al 2019  Nigeria | To determine the level of awareness, knowledge, and  practice of PCC and to identify factors influencing the  uptake and utilization of this care among women | Descriptive cross‑sectional study | pregnant women | 50 | 76% | 71.1% |  | Working as a civil servant  Having a tertiary education  Being a Yoruba  Parity ≤ 0  Married  Young adults |
| Ayalew et al 2017  Ethiopia | To assess women’s knowledge and associated factors in preconception care | Community based cross sectional study | Women | 422 |  | 31.8% | Young and middle adult  Educational status [Primary school, Secondary school, College and above]  History of family planning use |  |
| Boakye-Yiadom et al 2020  Ghana | to assess the awareness, knowledge, attitude and practice of pregnant women attending ANC | Descriptive cross-sectional study | women within the age range of 16  and 40 years | 200 | 34.5% | 42.5% = had poor knowledge  34% = moderate knowledge  23.5% =high knowledge on PCC. | Having a tertiary education  Being a Muslim | Young adult  Married |
| Al-Darzi et al 2013  Egypt | To measure the level of knowledge  about periconceptional use of folic acid  among pregnant women | Observational cross-sectional  study | pregnant women aged 18–45 years | 660 | 62.4% | 12.0% | Gravidity ≤2 | Young adult  Secondary education  Not working  Sufficient ANC visits in current pregnancy |
| Demissie et al 2019  Ethiopia | To assess the preconception care  utilization and determine factors that influence the  uptake | Community based cross-sectional study | reproductive age women | 410 | 35.4% | 17.3% =good  knowledge |  |  |
| Edalia 2022  Kenya | To determine the level of knowledge on preconception among women at the  reproductive health clinic | Descriptive cross-sectional study | women of reproductive age (from 15 to 49 years) | 224 | 19% | 42% |  |  |
| Ekem 2018  Nigeria | To assess the level of awareness and utilisation of PCC services. | Descriptive cross-sectional study | pregnant women | 450 | 42.2%=aware | 31.7%=had good knowledge | Young adult  Living in an urban area  Being in a tertiary education  Delivery in a health facility | Married  Parity ≤ 2 |
| Ezegwui et al 2008  Nigeria | To determine the awareness and practice of preconception care | Descriptive cross-sectional study | pregnant women | 1,331 | 43.1% |  | Having a post graduate education |  |
| Fekene et al 2020  Ethiopia | To identify the level of  women’s knowledge, uptake and associated factors of PCC | Community-based cross-sectional study | reproductive age women | 669 | 22.1% | 26.8%= had a good knowledge  73.2%= had inadequate knowledge | Working as NGO employee  Having completed grade 9–12, college and above  Having delivered in a health institution  Utilizing PNC service  Having used modern family planning use | Being a Housewife, Student, Gov’t employee and Private business |
| Fikadu et al 2022  Ethiopia | To assess knowledge of pre-conception health, its relation to planned pregnancy, parity, family planning use, and education among married  women | Community-based cross-sectional study | married women | 337 | 63.2%=aware | 55.2% | Being a market trade vendor  Having a primary, secondary education and above  Parity > 2  Having a history of family planning use  Planned pregnancy | Working as Civil servant and Day laborer |
| Gamshe & Demissie 2022  Ethiopia | To identify perinatal factors  affecting knowledge and utilization of preconception care among  pregnant women | Descriptive cross-sectional study | pregnant women | 331 |  | 68.6% =have good  knowledge 31.4% =  had poor knowledge | Attended PNC for previous delivery  Having a history of perinatal complication  Being informed about PCC in perinatal periods |  |
| Goshu et al 2018  Ethiopia | To assess women’s  awareness on preconception folic acid supplementation and  associated factors | Community-based cross-sectional study | reproductive age group women | 422 | 5.9% = had good  awareness |  | Having a primary, secondary education and above  Receiving $101 -105 monthly  Having a history of family planning use  Having a health problem |  |
| Kachiro et al 2022  Nigeria | To assess the awareness and  perception of preconception care among women | Descriptive cross-sectional study | women | 177 | 59.9% | 44.1 | Young adult  Being a civil servant  Having tertiary education  Parity between 2-3 | Being a Hausa  Still births ≤ 0  Preterm births ≤ 0  Abortions ≤ 0 |
| Kassa & Yohannes 2018  Ethiopia | To assess the  level of knowledge and associated factors towards preconception care among mothers who gave birth | Facility based cross sectional study | pregnant women | 580 |  | 20% =had good knowledge  80% =poor knowledge | Women’s and husband’s having educational level of high school and above  Living in an urban area  Receiving ≥3676.0-birr as a monthly household income  Having attended at least one ANC visit  Having a history of pregnancy Induced Hypertension  Having a history previous infant with macrosomia | Monthly household income of 1001.0–2000.0 birr, 2001.0–3675.0 birr] |
| Khonje 2017  Malawi | To explore knowledge, attitudes and practices of pregnant women on  preconception care | Descriptive cross-sectional study | pregnant women | 767 | 24.3% | 84%=no idea about PCC |  |  |
| Demeke et al 2024  Ethiopia | To assess knowledge and attitude towards  preconception care and associated factors among women  of reproductive age with chronic disease | Descriptive cross-sectional study | women of reproductive age | 844 |  | 55.6%= had good  knowledge | Having a primary, secondary education and above  Parity (1)  Received PCC counseling  Having a duration of disease ≥ 5 years | Parity ≤ 2 |
| Lemma et al 2022  Ethiopia | To assess knowledge of PCC and the associated  factors among reproductive-age women | Community-based, cross-sectional study | reproductive-age women | 414 | 35.5% | 17.1%= had good knowledge of PCC  82.9%=poor knowledge | Receiving >5000 ETB as monthly household income  Working as a daily labourer  Time to reach health facility ≥34 minutes (on foot)  Gravidity > 2  Having a history of congenital abnormality  Having a history of neonatal death  Having a history of contraceptive use | Being a government employee  Earning a monthly household income of ETB 1000-5000 |
| Msigwa 2021  Tanzania | To assess the knowledge and practice of preconception care among women attending  reproductive health clinics | Descriptive cross-sectional study | Reproductive women aged 17 to 49 years | 424 | 91% | 70% had good knowledge of PCC | Young adults  Midwives, doctors’ and media as sources  Not having financial support | Being in primary and secondary education  Married  Not employed  Received health education |
| Oketch et al 2021  Kenya | To identify the factors influencing preconception care  services among women of reproductive ag | Mixed methods | Women of reproductive age | 241 | 30% = had no idea of the PCC |  | Young adult  Married  Having a secondary education  Being a self employed |  |
| Olowokere & Owofadeju 2015  Nigeria | To determine the  level of awareness and knowledge of preconception  care | Descriptive cross-sectional design | Women | 375 | 63.5% | 65.3%= good knowledge  34.1%= poor knowledge | Having a tertiary education | Young adult |
| Tesema et al 2021  Ethiopia | To assess  knowledge of preconception healthcare and associated  factors: a study among mothers | Community-based cross-sectional study | Women of the reproductive age | 522 | 60.8% | 51.1%. | Having a primary, secondary education and above  Having a history of family planning use  Being a housewife  Having a history of neonatal death | Being a student  Not having a history of neonatal death |
| Teshome ta l 2020  Ethiopia | to assess the level of  knowledge of PC and associated factors among pregnant women | Community-based cross-sectional study | pregnant women | 623 |  | 21.3% =had good knowledge of PCC. | Women’s and husband’s having secondary education and above  Planned pregnancy  Having pre-existing illness | Living in an urban area  Being a teenager and young adult  Having a (TV/radio)  Using a long-acting family planning prior to recent pregnancy |
| Umar et al 2019  Nigeria | To assess awareness and perception of preconception care among women. | Descriptive cross‑sectional study | women | 131 | 20.61%= aware  79.38%= unaware |  | Being a Hausa  Being unemployed | Young adult  Having a format education  Parity ≥ 1 |
| Yohanees et al 2019  Ethiopia | To assess the levels and correlates of knowledge  and attitude of preconception care among mothers who gave birth at public hospitals | Facility-based cross-sectional study | women | 370 |  | 53%= had good knowledge | Having a primary education | Had a radio  Have health care providers’ relatives  Had pregnancy planned  Had community meeting related to PCC  Had health care providers friends  No formal education |
